# Supplementary material for: The Bioactivity of a Hydroxytyrosol-Enriched Extract Originated after Direct Hydrolysis of Olive Leaves from Greek Cultivars
Source: Molecules. 2024 Jan 6;29(2):299. doi: 10.3390/molecules29020299 (PMC10818913; doi:10.3390/molecules29020299)
Supplement: Supplementary file 1 [file molecules-29-00299-s001.zip › molecules-2770671-supplementary.pdf]

Table S1. Quantification analysis of the tail parameters of DNA damage using Comet assay in EA.hy926 cells, treated with 10 μg/mL and 40 μg/mL of DHOLE in the absence or presence of 250 μM H_2O_2. Data are presented as mean ± SEM, from 100 randomly selected cells/sample.

Treatments	Tail moment	Fold change of the Averages	Tail length	Fold change	%DNA tail	Fold change
control	0.42 ± 0.11	1.00	9.5 ± 1.17	1.00	1.58 ± 0.28	1.00
H_2O_2	12.63 ± 1.5	30.07	62.05 ± 4.8	6.5	11.8 ± 1.2	7.47
DHOLE 10 μg/mL	0.042 ± 0.01	0.1	6.14 ± 0.52	0.65	4.72 ± 1.3	2.99
DHOLE (10 μg/mL) + H_2O_2	0.77 ± 0.3	1.8	11.43 ± 2.0	1.2	6.42 ± 1.43	4.06
DHOLE 40 μg/mL	0.42 ±0.2	1.00	11.15 ± 1.75	1.17	0.27 ± 0.1	0.17
DHOLE 40 μg/mL + H_2O_2	3.2 ± 1.77	7.6	19.57 ± 3.32	2.06	3.5 ± 0.95	2.21

DHOLE: Direct Hydrolyzed-Olive Leaf Extract
